# The relevance of WHO injury surveillance guidelines for evaluation: learning from the aboriginal community-centered injury surveillance system (ACCISS) and two institution-based systems

**DOI:** 10.1186/1471-2458-11-744

**Published:** 2011-09-29

**Authors:** Anna M Auer, Teresa M Dobmeier, Bo JA Haglund, Per Tillgren

**Affiliations:** 1 Department of Public Health Sciences, Division of Social Medicine, Karolinska Institutet SE-171 77, Stockholm, Sweden; 2 Community Integrated Health Services, Interior Health, 37-450 Lansdowne Street, Kamloops, British Columbia, V2C 3G2, Canada; 3 School of Health, Care and Social Welfare, Mälardalen University, P.O. Box 883, SE-721 23 Västerås, Sweden

## Abstract

**Background:**

Over the past three decades, the capacity to develop and implement injury surveillance systems (ISS) has grown worldwide and is reflected by the diversity of data gathering environments in which ISS operate. The capacity to evaluate ISS, however, is less advanced and existing evaluation guidelines are ambiguous. Furthermore, the applied relevance of these guidelines to evaluate ISS operating in various settings is unclear. The aim of this paper was to examine how the World Health Organization (WHO) injury surveillance guidelines have been applied to evaluate systems operating in three different contexts.

**Methods:**

The attributes of a good surveillance system as well as instructions for conducting evaluations, outlined in the WHO injury surveillance guidelines, were used to develop an analytical framework. Using this framework, a comparative analysis of the application of the guidelines was conducted using; the Aboriginal Community-Centered Injury Surveillance System (ACCISS) from Canada, the Shantou-Emergency Department Injury Surveillance Project (S-EDISP) from China, and the Yorkhill-Canadian Hospitals Injury Reporting and Prevention Program (Y-CHIRPP) imported from Canada and implemented in Scotland.

**Results:**

The WHO guidelines provide only a basic platform for evaluation. The guidelines over emphasize epidemiologic attributes and methods and under emphasize public health and injury prevention perspectives requiring adaptation for context-based relevance. Evaluation elements related to the dissemination and use of knowledge, acceptability, and the sustainability of ISS are notably inadequate. From a public health perspective, alternative reference points are required for re-conceptualizing evaluation paradigms. This paper offers an ISS evaluation template that considers how the WHO guidelines could be adapted and applied.

**Conclusions:**

Findings suggest that attributes of a good surveillance system, when used as evaluation metrics, cannot be weighted equally across ISS. In addition, the attribute of acceptability likely holds more relevance than previously recognized and should be viewed as a critical underpinning attribute of ISS. Context-oriented evaluations sensitive to distinct operational environments are more likely to address knowledge gaps related to; understanding links between the production of injury data and its use, and the effectiveness, impact, and sustainability of ISS. Current frameworks are predisposed to disassociating epidemiologic approaches from subjective factors and social processes.

## Background

The capacity to design and administer injury surveillance systems has steadily developed over the past three decades. In part, this capacity is reflected in the broad range of injury surveillance systems (ISS), which now operate within various data collection environments such as emergency room departments, health care centers, community settings, and military operations. Yet regardless of the environment in which an injury surveillance system operates, these systems depend on and require organizational investment and commitment. Moreover, in due course such investments from a public health perspective are subject to monitoring and evaluation that considers various aspects associated with effectiveness and impact.

Public health surveillance is described as being a systematic, ongoing, and cyclical process involving data collection, analysis, interpretation, and dissemination of health information for action [1,2]. From a public health perspective, this definition places emphasis on ensuring that information and knowledge gained through surveillance are translated into action and ultimately improved health outcomes [3]. As a tool, injury surveillance is considered critical to supporting evidence-based decision-making. This aspect holds particular significance for the dissemination and use of injury data to assess and define priorities for action and to inform the development, implementation, and evaluation of targeted injury prevention programming [4].

ISS can be evaluated in different ways using epidemiologic and public health oriented approaches. Epidemiologic approaches that are primarily data focused are critical to ensuring that the quality of data being collected serves as a credible source of information with defined limitations. Public health approaches to evaluation, are essential to ensuring that the reliability of a system is supported through assessments of the capabilities and functions of a system. These different approaches, each of which employs various evaluation methods and metrics, are being used to evaluate ISS. These approaches, however, have originated from sources intended to evaluate a range of public health and communicable disease surveillance systems [5-7].

In the absence of standardized guidelines specific to the evaluation of ISS, the World Health Organization (WHO) injury surveillance guidelines have provided a point of reference for undertaking evaluations. Published in 2001 by the WHO and the US Centers for Disease Control and Prevention (CDC) as a resource manual, components of the guidelines include background information about the problem of injury and an introduction to injury surveillance. Subsequent information outlines the attributes of a good surveillance system, steps to establish and maintain a system, and direction on the monitoring and evaluation of ISS [8]. The applied relevance of these guidelines, however, for ISS operating in various settings is unclear.

In 2008, a case study involving an evaluation of the Aboriginal Community-Centered Injury Surveillance System (ACCISS) from Canada provided the basis from which the relevance of the WHO injury surveillance guidelines could be examined in relation to the intended use of injury data being collected at a local level [9]. The ACCISS case study also provided a unique opportunity to consider ambiguities associated with the WHO guidelines from a public health perspective.

Furthermore, since institution-based ISS have also used the guidelines for evaluation purposes the opportunity to compare the application of the guidelines by the ACCISS with those of two additional case studies was undertaken. These additional cases were based on earlier reported studies and involved the Shantou-Emergency Department Injury Surveillance Project (S-EDISP) from China [10] and the Yorkhill-Canadian Hospitals Injury Reporting and Prevention Program (Y-CHIRPP) imported from Canada and implemented in Scotland [11].

The aim of this paper was to examine how the WHO guidelines have been applied in evaluating ISS in three different contexts. ISS attributes used by the WHO to define a good surveillance system, as well as evaluation methods outlined in the guidelines were key components utilized to develop an analytical framework. Using this framework, the application of each respective component was examined by considering two key questions: (1) how relevant are the WHO guidelines for evaluation at a community level? and (2) how relevant are the WHO guidelines for institution-based injury surveillance systems? As attention shifts towards evaluating the operation, effectiveness, and ultimately the value of ISS; the development of evaluation frameworks, used as tools to organize the criteria and methods by which evaluations are undertaken require critical consideration. The study of evaluation related ambiguities will provide a needed space for deliberation in the development and use of ISS evaluation frameworks to support findings that are valid, reliable, and useful.

## Methods

### Analytic approaches

In this qualitative multi-case study, three main steps were used to conduct a comparative analysis of the WHO injury surveillance guidelines. The first step involved the development of an analytical framework [12]. The framework was developed using the attributes of a good injury surveillance system (i.e. simplicity, flexibility, acceptability, reliability, utility, sustainability, timeliness, and security and confidentiality) as identified in the guidelines. In addition, the instructions for conducting retrospective, process, and system environment evaluations of ISS were assessed to identify the primary purpose and evaluation methodology suggested for each type of evaluation for inclusion in the analytical framework. The attributes of a good surveillance system and types of evaluation were then considered in conjunction with the four inter-related activities of surveillance (i.e. data collection, analysis, interpretation, and dissemination). The second step involved deconstructing case study information to establish comparative units of analysis [12,13]. Available segments of descriptive data were extracted to identify the primary characteristics associated with each ISS relative to the type, age, scope, focus, setting and design, locus of operational management, and end-users. Additional data segments, which identified the focus and methods, associated with each ISS evaluation were also extracted. The third step entailed examining the application and relevance of the guidelines considering similarities and differences associated with the operational aspects of each system. This examination first considered community-based lessons drawn from the ACCISS and then S-EDISP and Y-CHIRPP; specifically taking into account the context of each case study. All data extraction was consistently conducted by a single author (AMA) using the analytical framework developed in step one. Data extractions were then reviewed by each author independently and consistency was determined through consensual analysis [14].

Background information and analysis related to the ACCISS case study was based on the project's evaluation report as well as the knowledge and experiences of (AMA and TMD), the paper's first two authors, with the Secwepemc Nation Injury Surveillance Project. These authors were involved in supporting the implementation of the ACCISS through on-site community visits and subsequently formed part of a five-member evaluation team comprised of three external evaluators and the authors as internal evaluators. The team's external evaluators assumed primary responsibility for data collection, which encompassed a document review, focus groups, and individual interviews. Information and analysis related to the two additional case studies relied on evaluations reported in the published literature. These case studies were selected based on clear references to the use of WHO guidelines as elements of an evaluation framework.

### ACCISS case study background and setting

In Canada, access to health data by community-level service providers presents challenges in that available data often falls short of its capacity to inform targeted programming initiatives. For Aboriginal communities health data challenges are uniquely distinct in that they are historically linked to the legacy and impacts associated with "forced acculturation and failed assimilation policies" [15]. In 1939, Aboriginals were brought under the responsibility and jurisdiction of the federal government with services in the area of health, education, and social services being administered through provincial and territorial health services [16]. Historically, the federal government has collected and controlled data on behalf of Aboriginal people. As a result, Aboriginal people have been faced with fundamental issues such as lack of control and access to their own health data as well as significant limitations associated with data quality, reliability, and ultimately community-based relevance and usefulness [17].

Within this historical background, the ACCISS was designed by First Nations for First Nations to ensure cultural relevance and acceptability in support of "building capacity for self-determination" [18]. Central to the capacity-building focus, the system was designed: to facilitate community management of the key activities associated with injury surveillance; and to ensure active use of the data. Accordingly, the ACCISS represents a local level ISS designed to operate within community environments that are diverse with respect to population, size, geographic location, health service delivery, community infrastructure, resources, and administrative management [9].

In 2005, ten of seventeen Secwepemc Nation communities, located in the province of British Columbia, launched the Secwepemc Nation Injury Surveillance Project and initiated community-centered data collection using the ACCISS. British Columbia is the westernmost province of Canada, located on the Pacific coast of North America. It is the third largest of Canada's ten provinces with a land area of 944, 735 km^2 ^and a population of approximately 4.1 million [19]. Geographically, Secwepemc Nation bands are spread across 18% of the total area of the province. About one third of Aboriginal peoples in Canada comprised of First Nations, Métis, and Inuit live in British Columbia. The collective provincial population of these three groups of Aboriginal peoples approximates 196, 000 of which 129, 000 are First Nation [19]. First Nation communities in the province represent over 200 culturally heterogeneous communities. The project communities, a reflection of this diversity, vary considerably in population, size, geographic location, health service delivery, infrastructure, resources, administrative management, distances to town centres, and access to acute care and rehabilitative health services. The total estimated population of the 10 project communities is 3800 residents with community specific populations ranging from 100 to 1000 residents [9].

Each of the ten communities initiated data collection based on its readiness to do so. As a result, data collection start dates were staggered over a 16-month period[9]. At the time of the evaluation all project communities had been collecting data for a minimum of twenty-two months [9]. In 2007, an evaluation was undertaken for the purpose of identifying lessons learned related to the implementation of the project. One key objective within the overall evaluation focused on assessing the capabilities and usefulness of the ACCISS.

### ACCISS data management components and processes

The ACCISS uses three data management tools that consist of a paper-based injury surveillance form, a data-entry module, and a data analysis module. The system is based on a minimal dataset approach and collects data on all types of intentional and non-intentional injuries. The electronic components or modules of the ACCISS use Epi-Info as the software platform to drive data entry and analysis functions. Each injury case captured on an injury surveillance form is entered and compiled electronically using the data-entry module. Subsequent analysis is conducted using the data analysis module.

The number of data sources and data collectors varies from community to community. Typically, injury data are being collected through a network of community-based services such as day care facilities, health centres, schools, Elders facilities, and home and community care programs. Community-centered networks are congruent and unique to the infrastructure of each community. Injury surveillance forms are completed by staff within the network. Completed forms are then forwarded to designated staff for data entry and analysis. Designated staff, based at the health centres or societies serving project communities, assume day-to-day responsibility for collecting, coordinating, and monitoring the data collection activities of the network. Staff also assume delegated responsibilities related to data analysis, interpretation, and dissemination. The identification of injury cases and actual data collection relies predominantly on members of the data collection network and designated project staff. The identification of injuries requiring hospitalization or resulting in death depend on designated community staff maintaining channels of communication with acute care and rehabilitative health services staff to identify and flag injury cases involving community members.

### Evaluation background of community and institution-based systems

The ACCISS, centered as an ongoing community-based injury surveillance and prevention program involving ten project communities, had an operational period of two years at the time of evaluation. All ten-project communities of the Secwepemc Nation program were involved in the evaluation [9]. The S-EDISP in China [10], an institution-based system, was identified as a being part of a larger injury surveillance study involving five hospitals with an operational period of approximately one year [10]. The evaluation of the S-EDISP, also an institution-based system, was limited to one hospital representing the largest hospital in the study. The Y-CHIRPP, a system imported from Canada and implemented in Scotland, operated for a ten-year period prior to being discontinued [11]. The operation and evaluation of the Y-CHIRPP involved one site, the largest children's hospital in Scotland.

## Results

**Figure 1:**
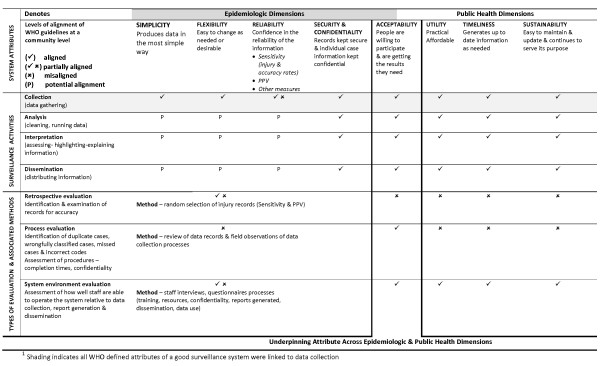
**ACCISS evaluation and relevance of WHO guidelines at a community level**.

**Figure 2:**
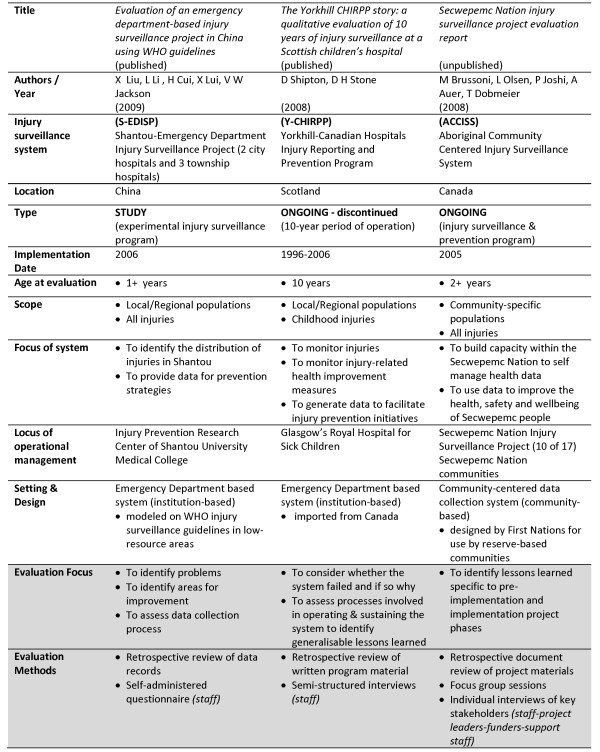
**Interrelationship of basic ISS characteristics to evaluation**.

In this section, results addressing this study's two research questions are reported sequentially. The results begin with findings associated with examining the relevance of the WHO guidelines at a community level, using the ACCISS case study in Canada (Figure 1). Subsequently, results associated with comparing the relevance of the guidelines to the ACCISS with two institution-based data gathering systems utilizing the S-EDISP in China, and the Y-CHIRPP in Glasgow are presented (Figure 2).

### Relevance of attributes at a community level

At a macro level, the guidelines relate to and align with the nature of the environment in which the ACCISS is intended to function as well as how the system was initially developed and subsequently adopted for implementation by First Nation communities. Juxtaposing ISS attributes and the types of evaluation with the four inter-related activities of surveillance found that the guidelines gravitate towards an epidemiologic perspective. At the same time, the juxtaposition highlighted the potential alignment and use of these components for application at a community level when considered from a public health perspective (Figure 1). 

At a meso level, the WHO defined attributes of a good surveillance system, fluently and consistently link to some aspect of the data collection process. Furthermore, unless juxtaposed across the four inter-related activities of surveillance, the attributes predominantly focus on epidemiologic issues and methods (Figure 1). In particular, the attributes of simplicity, flexibility and reliability focus attention on the nature and quality of ISS instrumentation as well as the quality and reliability of injury data being collected. The attribute of reliability also distinctly brings attention to epidemiologic issues addressing the completeness of each record, accuracy in relation to correct coding and classification of data as well as the representativeness of data being collected. Further, specific methods are described to calculate and quantify reliability based on measures of sensitivity and positive predictive value (PPV), respectively addressing the degree to which injury events are detected and non-injury events are excluded from a system. In addition, security and confidentiality emphasize the importance of ensuring the prevention of harm to individuals through the safekeeping of records and information gathered.

At a micro level, the relevance of the WHO defined attributes of a good surveillance were examined in relation to the age and stage of the Secwepemc Nation injury surveillance project. Accordingly, the epidemiologic dimensions were assessed with a primary focus on data collection while public health related dimensions were linked to data analysis and interpretation. Based on these foci, evaluation data revealed important interrelationships between the attributes of a good surveillance system and each respective surveillance activity. For instance, simplicity from an end-user perspective considered whether activities related to analysis, interpretation, and dissemination were sufficiently straightforward to ensure that these activities could be undertaken by community-based staff. Similarly, flexibility considered data collection tools and processes as well as the ability to tailor data analysis, interpretation, and dissemination functions to the needs and interests of each project community. This pattern was evident across attributes.

Acceptability as an attribute considers the willingness of individuals to participate in the system as well as whether results are being achieved. From this viewpoint, the attribute of acceptability encompassed data collection as well as a range of additional factors when considered in relation to data analysis, interpretation, and dissemination. The relevance of acceptability in relation to the ACCISS was linked to numerous factors, each of which consistently interrelated to each respective attribute of a good surveillance system and surveillance activities. Some of these factors included: the affordability of the data management tools; positive hands-on learning experiences associated with seeing and working with injury data; the capacity to self-generate reports; the ability to produce data that was considered relevant in the day-to-day work of staff; and the capacity of each project community to independently respond to emerging injury issues.

Lastly, utility, timeliness, and sustainability focus primarily on the practicality of procedures, budget, the ability to generate timely information, and system maintenance placing emphasis on staff and the supporting agencies actively involved in performing data related functions. Attention to data dissemination, the use of knowledge, and factors related to sustainability is notably limited in the WHO guidelines. Accordingly, these attributes shift the balance of attention towards data management and away from data dissemination and end-users needs. The relevance of these latter attributes to the ACCISS was consistently and closely connected with meeting Secwepemc Nation end-user needs and program goals. From an administrative viewpoint, the attributes of utility, timeliness, and sustainability of the ACCISS were identified as important considerations in the decision to implement the system. Furthermore, the necessity and interest of the project communities to strategically direct the use of limited resources was closely linked to overall acceptability of the system for implementation.

### Ambiguity of recommended types of evaluation

The application of the three recommended types of evaluation presented challenges. Initial interpretations of this component were hindered by the choice of terminology and general lack of conceptual clarity. Despite this lack of conceptual clarity, two of the three recommended types of evaluation were notably consistent with an epidemiologic perspective. Retrospective evaluation was described in the guidelines as a process of looking back at injury records, using random selection strategies to examine and assess sensitivity and PPV. This description, focuses attention on epidemiologic issues, and typifies evaluation methods. The second type of evaluation, referred to as a process evaluation, was described as involving the direct observation and assessment of data records and data collection processes. This contracted explanation typifies evaluation methods with an epidemiologic focus rather than a comprehensive process evaluation. The third type of evaluation, a system environment evaluation, was described as assessing how well staff are able to operate the system. This description, which theoretically relates to looking at ISS operations and operational environments, was interpreted as being more representative of a category of factors to be considered for evaluation rather than a type of evaluation. This third type of evaluation was identified as being more consistent with a public health perspective.

### Potential misalignment of guidelines for community-based application

Terminology aside, the attributes of a good surveillance system as defined by the WHO, types of evaluation, and associated methodologies, highlighted three areas of potential misalignment for community-based application. First, the attributes at a community level are subject to local interpretation and contingent on locally defined processes. Although the attribute of reliability, was identified as a very important construct for the Secwepemc Nation project communities, it was correlated with ensuring that data collection networks adequately reported and represented injury cases relative to each community population. As such, the efficacy of using sensitivity and PPV as metrics of reliability at the community level was determined to be very low by the ACCISS evaluation team. Based on the ACCISS the measures of reliability, outlined in the guidelines, represented a gap for community-based relevance and application (Figure 1).

In addition, the capacity building focus of the ACCISS centers attention on each of four inter-related injury surveillance activities of data collection, analysis, interpretation, and dissemination. As such, each attribute requires consideration relative to each of these four key activities. Moreover, since the use of injury data for injury prevention is central to the Secwepemc Nation project communities, the attributes of acceptability, utility, timeliness, and sustainability assume a higher level of relevance from a public health perspective. Overall, the relevance and interpretation of each of these attributes was driven by and linked to the end-users of the ACCISS.

Second, typical methodologies associated with an epidemiologic perspective are likely to be misaligned with community-based operational settings including culturally related factors (detailed in a separately drafted manuscript). One key factor related to the feasibility of evaluation methods for the ACCISS considered the number of injury cases documented relative to given time-periods, as well as the number and location of data collection sites and networks across a vast geographic territory. Given that the number and pattern of documented injury events varies considerably in relation to the size, location, and seasonal activities of each project community, field observations would have necessitated either multiple site-visits or residing in the communities for extensive periods. Issues of practicality, time and available resources outweighed any significant benefits.

At a community level, ACCISS data quality linked to missing or incomplete data, missing or duplicate cases and coding, is assessed and validated by way of a systematic and periodic audit of injury records by each project community. Audits were consistent with methods associated with the retrospective examination of records, however, simplified assessment criteria, standards, and baseline measures were being used for ongoing monitoring. From a cultural perspective, data collection involving third party observations of staff interviewing injured community members would have been intrusive and breached community-developed protocols and policies established to ensure confidentiality and anonymity. Evaluation methodologies as outlined in the WHO guidelines were only partially aligned with the operational environment of the ACCISS and represented a gap for community-based relevance and application (Figure 1).

Third, the relevance of the guidelines required consideration in view of the age and stage of the ACCISS as a program within the project communities. The nature and level of attention to the epidemiologic dimensions of the attributes shifted during pre-implementation and implementation stages. More time and emphasis was placed on monitoring the epidemiologic dimensions of the attributes during the early stages of implementation and less time afterward as data collection activities became more established. As data collection activities stabilized and became more routine, attention increasingly shifted towards the public health dimensions of data analysis, interpretation, and dissemination to promote use of injury data by end-users. This was reflected in the ACCISS evaluation, which identified early successes associated with the applied use of injury data. Project communities were using their data to engage community members and to address environmental risk factors identified in the community. This was found to be consistent with the high value that project communities placed on having community activities driven and informed by community data and represented a critical aspect related to overall acceptability of the system.

The application of the WHO guidelines for evaluation at a community level was enhanced by considering the attributes of a good surveillance system from the viewpoint of end-users in relation to the: goals and objectives of the ISS across inter-related injury surveillance activities; local operations and environments; as well as the program implementation stages of the ACCISS. Further, the attribute of acceptability when considered from the point of view of stakeholders and end-users served as an underpinning attribute across epidemiologic and public health dimensions (Figure 1). This facilitated balanced attention between data management issues and public health interests from an end-user perspective.

### Relevance across different operational environments

The aim of reviewing two other ISS evaluations in relation to the ACCISS was to explore the application of the WHO guidelines across different operational settings and evaluation foci. The analysis found that although ISS are connected by a common interest to contribute to injury prevention and injury surveillance functions that basic characteristics across ISS and evaluation needs can vary significantly in relation to the location, scope, age, and locus of operational management of a system (Figure 2).

The S-EDISP in China and the Y-CHIRPP in Scotland were both Emergency Department and institution-based systems [10,11]. The former encompassed multiple data collection sites gathering data on all injuries while the latter ISS was comprised of a single site collecting injury data specific to children. The ACCISS, as a community-based ISS, collected data on all injuries involving community members and non-residents injured within community boundaries. Like the S-EDISP, data collection involving the ACCISS encompassed multiple sites and locations. The S-EDISP was administered by a research center, the Y-CHIRPP by a hospital dedicated to the treatment of children, and the ACCISS by a First Nations Health Directors program team.

### Relevance of age and status across ISS

Unambiguously, the focus of each evaluation was linked to the age and status of the ISS at the time each evaluation was conducted. Correspondingly, the application and interpretation of the WHO guidelines varied in relation to the age of each system. Conversely, interpretations of the guidelines were correlated with the level of attention and importance allocated to managing data collection, analysis, interpretation, and dissemination functions.

The S-EDISP evaluation focused on epidemiologic issues primarily centered on data collection activities [10]. The WHO defined attributes of a good surveillance system, as markers of performance focused predominantly on the feasibility of data collection. Reported results were concentrated on the system's instrumentation, case identification, data collection processes, and factors influencing data collection. Data analysis and interpretation functions were noted only briefly as being carried out by trained research staff while data dissemination was limited to a discussion about responses generated from a single question included on a self-administered staff questionnaire.

The ACCISS evaluation focused on pre-implementation and implementation phases of the project incorporating both epidemiologic and public health perspectives [9]. The attributes of a good surveillance system considered all key ISS functions from the viewpoint of the system's key stakeholders and end-users. Reported results were centered on project planning, implementation, ISS management and project outcomes as well as the capabilities and usefulness of the ACCISS in relation to data collection, analysis, and interpretation. Data dissemination functions were in the early stages of development at the time of evaluation.

The retrospective evaluation of the Y-CHIRPP, focused on an extensive program period from the program's inception to its conclusion [11]. Like the ACCISS, the Y-CHIRPP evaluation considered both epidemiologic and public health perspectives. The public health perspective emerged by way of an examination of the strengths and weaknesses associated with the steps of developing an ISS. Key individuals involved in these steps over the Y-CHIRPP program period informed this examination. Closely aligned with the four key functions of surveillance, reported results highlighted a significant and weighted emphasis on data collection with little to no emphasis directed towards data analysis, interpretation, and dissemination.

### General relevance of guidelines across ISS

At a macro-level, given the general purpose and functions associated with ISS, the guidelines demonstrated a level of universal relevance for evaluation across three diverse operational environments. At a meso-level, the significance of the guidelines was less categorical. The relevance of the guidelines appeared contingent on the interpretation and use of the guidelines in relation to the age and status, locus of operational management, and design of the system. At the micro-level, relevance was dependent upon promoting congruency between performance measures and methods and system specific characteristics, processes, and operational environments.

### Relevance of ISS characteristics to evaluation frameworks

System specific characteristics informed and influenced the focus and methods of evaluation associated with each ISS that was studied. The varied interpretation and application of the guidelines underscored the scope of considerations that can emerge relative to levels of multiplicity, acceptability, locus of operational management and differing perspectives.

Levels of multiplicity associated with ISS characteristics were evident within and across systems, in relation to such basic traits as the number of sites and partners associated with a system, number and type of individuals engaged in key surveillance functions, and system and site-specific structures and processes. These variations, whether community or institution-based, give emphasis to the symbiotic nature of ISS characteristics relative to each operational environment. At the same time, the multiplicity of ISS characteristics brings attention to how differently evaluation metrics are being interpreted and applied across diverse systems and settings.

### Acceptability as an underpinning attribute

Acceptability, interpreted and conceptualized more broadly as an attribute underpinning both epidemiologic and public health perspectives, further highlights a range of inter-related ISS factors to be considered. Acceptability conceptualized in this way, in relation to the S-EDISP, Y-CHIRPP and ACCISS, was linked to a range of factors. Factors related to staff included: staffing levels and workload; staff support, motivation and perceptions; time limitations; and levels of training. Factors related to injury surveillance functions included: confidentiality; operating costs; levels of complexity and resources associated with each injury surveillance function; and the capacity to tailor and manage flexible processes [9-11]. Other key factors were related to: the ability to evaluate the reach and use of data collected by ISS; leadership support and continuity; involvement in decision-making processes; visible successes; measureable achievements; and the capacity to recognize, involve, and meet end-user needs [9-11].

### The influence associated with the locus of operational management

In addition, the locus of operational management emerged as an important ISS characteristic influencing the development, management, monitoring, and evaluation of a system. In line with this characteristic, arise evaluation issues related to the historical background and origins of a system and the basis for its establishment. This background inherently inter-connected with factors related to leadership, organizational structure, mandate, and authority linked to the operational management of a system. Additional inter-related factors associated with the locus of operational management included: decision-making and accountability processes and structures; capabilities to manage all four key functions of injury surveillance; and the capacity to manage and coordinate collaborations involving partners and end-users [9-11]. In a related manner, the compatibility and deployment of epidemiologic and or public health perspectives appears contingent on operational environments.

The scope of issues identified relative to the evaluation of ISS is significant and the standard application of measures and methods of evaluation present challenges across different operational settings. Systematic attention to evaluation issues and methods, however, may support a starting place for re-conceptualizing evaluation frameworks.

## Discussion

### Evaluation paradigms

The key finding identifies that the evaluation components of the WHO guidelines are disproportionately focused on data collection instruments and injury surveillance functions largely associated with data collection. Although this focus contributes to ensuring quality data, at the same time it deflects attention from the dissemination and use of information by end-users central to a public health perspective. The disproportionate epidemiologic focus is rooted in the origin of the guidelines. At a time when many small resource-constrained countries were challenged to establish ISS the primary focus of the guidelines was to support the establishment rather than the evaluation of ISS [20,21]. The differing interpretations and application of these guidelines, however, are likely to perpetuate and reinforce a predominantly epidemiologic perspective unless alternative evaluation paradigms are explored and conceptual clarity is developed.

An epidemiologic perspective places emphasis on the quality of health data and standards of data collection and analysis. This focus aligns well with systems designed for accurate and effective reporting and monitoring of health related events and diseases. In addition, the epidemiologic focus aligns well with establishing discrete definitions, standardized evaluation metrics, and quantifiable measures of performance. As such, epidemiologic indicators more readily align with the use of the SMART criteria, associated with targeted goal setting and of task performance through measures that are specific, measurable, appropriate, and time-bounded [22].

If, however, the fundamental benchmark of a good ISS is linked to the notion of 'data being linked to preventive action', then the perpetuation of a predominantly epidemiologic evaluation paradigm will fail to inform us of the conditions and processes necessary to achieve and sustain this standard. From a public health perspective, evaluation indicators developed to consider the importance of place, settings, and context can help to reconceptualize evaluation paradigms for ISS. The ACCISS as implemented by the Secwepemc Nation is regarded as a capacity-building tool that supports self-determined data management and injury prevention programming. Within this program context the ACCISS is understood to function in four ways: first as an epidemiologic tool; second as a pathway that supports capacity-building to undertake injury surveillance and health promoting and prevention activities; third, as a mechanism to support collaboration and partnerships in health; and fourth as a program initiative fundamentally focused on reducing the burden of injuries.

The evaluation of the ACCISS considered the system's primary end-users, program goals, objectives, and intended program outputs. In relation to these key ISS characteristics, the evaluation then examined: facilitating factors and challenges relative to the stage of the project and injury surveillance functions; factors related to community readiness to implement the ACCISS; and partnership interests and support roles that were considered important to program stability and sustainability but removed from day-to-day activities and actual injury surveillance functions [9]. As such, the overall evaluation framework for the ACCISS balanced both epidemiologic and public health perspectives based on an alternative and culturally holistic evaluation paradigm.

### Conceptual clarity and the four basic functions of injury surveillance

Further, the conceptual clarity related to the evaluation components of the WHO guidelines was found to be lacking. Although the definition of health surveillance is widely adopted, information examined in relation to the S-EDISP, Y-CHIRPP and ACCISS would suggest that different interpretations and assumptions exist in relation to the four basic functions of injury surveillance. In varying degrees, there appears to be a common presumption of understanding as to what each of these activities entails and consequently how these activities are carried out, when in reality these functions can vary significantly by the structure and operational environments in which ISS exist.

Beginning at the end of the injury surveillance cycle, data dissemination infers that data gathered, once analyzed and interpreted will be documented and communicated. Linked to the definition of health surveillance is the dissemination of information for action, inferring more active than passive communication processes. The definition also introduces the concept of knowledge translation, which involves an interactive exchange of information between those who produce the information and those who use the information [23]. Reasonably, the goals and activities related to active and passive mechanisms of data dissemination will vary considerably based on the wide range of operational environments and the local, regional or national scope of systems. Given the diversity and scope of ISS, data dissemination goals and objectives of ISS should be well defined, explicitly stated, and at the forefront of any planning and development processes.

Data analysis and interpretation functions also require conceptual clarification as descriptions of these functions also vary. Some descriptions appear to combine the activities of data analysis and interpretation, again leaving open to interpretation how these functions are being carried out. Although it is understood that data analysis precedes interpretation, approaches to these functions are generally unique to and aligned with the basic characteristics of an ISS. Moreover, the issue of data accessibility by end-users in relation to these functions is often poorly delineated or addressed.

As a study or pilot ISS project, like the S-EDISP, data analysis and interpretation functions may be undertaken by study personnel, while larger systems may rely on a core group of staff or external sources of technical expertise which in turn may or may not involve end-users. The ACCISS relies on designated staff to undertake data analysis functions while data interpretation also includes community-based staff and the Health Directors to ensure that data are considered in the context of the project communities. Further, dialogue related to data interpretation is linked to injury prevention planning. In the case of the ACCISS, the data interpretation function also serves as a system specific method to monitor issues related to reliability.

### ISS standards and models

With respect to data collection, many systems center this function within the domain of health care settings and rely on trained health care professionals to identify and collect information on injury cases. This profile, however, should not be confused or presumed to be a preferred standard for ISS but rather a model by which an ISS can operate. The ACCISS model, involves a network of data collectors of varied service providers occupying professional and non-professional roles within participating project communities. In complete contrast to the ACCISS model, an injury surveillance system for violence such as the US National Violent Death Reporting System (NVDRS) operates as a relational database that links various data sources. Within the NVDRS model, data collection essentially entails establishing data linkages to existing data sources without creating or collecting new primary data [24]. The scope of these variations underscore the critical importance of delineating how basic injury surveillance functions are carried out, and aligning relevant evaluation methods and measures to the context and operational environment of the system. Ultimately, examining and understanding the dynamic interrelationship of system specific characteristics will provide a basis from which our understanding of effective ISS can develop.

### Conceptual clarity and the attributes of acceptability and sustainability

Although the effective use of data, acceptability, and the sustainability of ISS represent primary concerns, clarity relative to how these aspects should be considered and evaluated remain relatively unfocused and unaddressed. The WHO ISS guidelines bring attention to these aspects, as do CDC's updated guidelines for evaluating public health surveillance systems [5], however, the significance and interpretation attached to these aspects remains inadequate. In its application, acceptability as an attribute of a good surveillance system, presents as a basic example of this inadequacy. The development of an ISS in the Caribbean, described acceptability as being "more often a proxy for system simplicity" [25] and subjective in nature. Mitchell et al, who outline the development of a prototype ISS evaluation framework promoting data quality, note that acceptability as a characteristic "was excluded because of disagreement about its definition despite being rated high in importance" [26].

Further, acceptability as described in the WHO guidelines does not explicitly address cultural relevance, yet several key aspects important to First Nations were found to be connected with acceptability. These aspects related to the: system working well within varied community populations and geographic environments; adaptability of the system in the face of varied skill sets; and data collection networks tailored to operate in relation to community-specific infrastructures. Another critical factor was the central importance and application of principles related to ownership, control, access, and possession, otherwise known as OCAP principles [27]. Acceptability was transparently linked to the cultural relevance of the ACCISS. As developers, owners, managers, and end-users of the system, First Nation communities can self-determine action on injury priorities by linking their population specific data to planning. Culturally related factors, particularly relevant to public health, have also been reported in relation to the Jamaican injury surveillance system and the S-EDISP [10,28,29].

### Relevance of the locus of operational management

Also emerging from the comparative analysis of the WHO guidelines, is the central importance of staff, stakeholders, and end-users roles, and the relevance of the locus of operational management to ISS. These aspects correspond to lessons learned associated with the development and evaluation of ISS. During pilot testing of the Y-CHIRPP, staff resistance was noted as an implementation challenge and after a ten-year program period, the same factor warranted similar attention [11,30]. Both successes and challenges link back to staff, stakeholders, and end-users in relation to the locus of operational management. Early involvement and coordination, physical proximity, and mechanisms to link with end-users are common facilitating factors across diverse operational environments [31-35]. Reasonably, it follows that the locus of operation assumes significance in terms of its capacity to undertake leadership and coordination functions relative to its own structure and organizational mandate. This suggests important and dynamic relationships between leadership and coordination functions and the locus of operational management, the cyclical activities of injury surveillance, and end-users.

### Cyclical level of importance of attributes

Another observation, in relation to the WHO attributes of a good surveillance system, is that the attributes may have cyclical levels of importance over time. Simplicity and flexibility may have more relevance during the development and pilot testing of a system while the attribute of reliability may have a higher degree of significance as an ongoing or periodic measure of data quality and performance. In a similar manner utility, timeliness, and sustainability may also assume different degrees of priority relative to the longevity of a system. In the same way, if one reasonably assumes that utility is a non-static trait then utility should be monitored on a periodic basis in relation to a system's longevity and evolving end-user needs. The cyclical levels of importance further emphasize the significance of considering the attributes in relation to the age and status of an ISS.

### The interrelatedness of ISS characteristics

Using components of the WHO guidelines to develop an analytical framework enabled a systematic assessment of relevance in relation to the different operating environments of ISS. The identification of key ISS characteristics associated with all three systems enabled a comparative analysis of how the guidelines had been interpreted and applied in the context of different operational environments, developmental stages, key injury surveillance functions, and system specific attributes. The comparative analysis also facilitated reflections relative to the interrelatedness of ISS characteristics and levels of congruency with existing evaluation methods and metrics.

### ISS operational environments and evaluation

**Figure 3:**
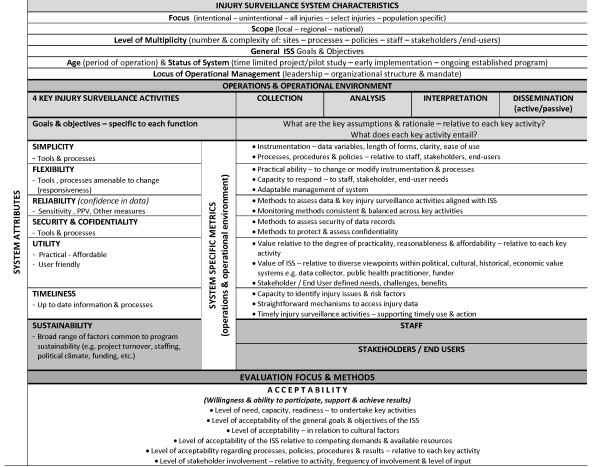
**ISS evaluation template**.

As an alternative evaluation paradigm, key evaluation factors and findings identified through the comparative analysis were synthesized in an evaluation template for ISS (Figure 3). The focus of the template is to reframe the praxis of how evaluation issues and metrics can be considered. The template reconceptualizes aspects of the WHO injury surveillance guidelines from a public health perspective. Data collection, analysis, interpretation, and dissemination activities were considered vis-à-vis system specific operational environments and the WHO guidelines.

The template centers the characteristics of an ISS as guiding determinants of an evaluation framework strategically grounded by the perspectives of staff, stakeholders, and end-users. If the benchmark of ISS is 'data being linked to preventive action' then the focus, scope, level of multiplicity, general goals and objectives, age and status of the system, and locus of operational management should consistently relate to its staff, stakeholders, and end-users. In this way, the template is intended to bring systematic attention to important considerations that link system attributes with subjective factors and social processes. In addition, ISS characteristics are positioned in the template as primary indicators to guide the development of system specific metrics to ensure relevance and alignment with different operating environments.

The template, also positions the WHO attributes of a good surveillance system across all four-injury surveillance functions to expand the conceptualization and relevance of each attribute. Each attribute listed in the left hand column is considered in relation to data collection, analysis, interpretation, and dissemination represented horizontally as a continuum composed of four distinct yet inter-related injury surveillance activities.

Acceptability as an attribute of a good surveillance system is purposefully situated as an underpinning attribute to methodically bridge all key aspects of the system in relation to its purpose, functions, age, and status. Acceptability is also viewed as being essential at macro, meso, and micro levels that link back to functions of leadership and coordination and the locus of operational management. Similarly, acceptability relates to the adequacy and relevance of an evaluation and evaluation methods in relation to the needs and interests of the system's staff, stakeholders, and end-users.

Lastly, the attribute of sustainability is positioned towards the base of the template to bring attention to the important and dynamic inter-relationships to be considered between a broad range of program factors and those most connected to the ISS. The overall configuration of the evaluation template is intended to support the examination of interrelationships that link epidemiologic dimensions with ISS characteristics, operations and operational environments, and social processes.

Case studies are subject to criticism with respect to generalizability, however, the use of multiple cases can strengthen and support generalizations [36,37]. In this comparative analysis, the examination of cases grounded in the reality of program implementation supported reflections related to the interpretation, relevance, and application of the WHO guidelines. By looking at similarities, differences, patterns and interrelationships across more than one case, lessons learned have the potential to be conceptualized and transposed beyond the boundaries of a single case study. The further development of evaluation frameworks would benefit from an examination of the grey literature as the published literature on ISS evaluations remains limited.

## Conclusions

Dynamic and contextual factors are central to a public health perspective. As such identifying and understanding the interrelationship of contextual factors relative to injury surveillance takes on critical importance. If a fundamental principle and benchmark of injury surveillance is to ensure that knowledge gained through surveillance is translated into action then evaluation frameworks based predominantly on an epidemiologic perspective will need to be re-conceptualized. The WHO guidelines over emphasize epidemiologic attributes and under emphasize public health and injury prevention perspectives that require adaptation for context and culturally based relevance.

Findings from this study suggest that it is insufficient to conduct ISS evaluations based predominantly on an epidemiologic perspective. Furthermore, the findings suggest that attributes of a good surveillance system when used as evaluation metrics, cannot be weighted equally across ISS. Acceptability as an underpinning attribute of a good ISS may hold more relevance than previously recognized especially in relation to organizational investments, effectiveness, and impact. Structured and systematic approaches are needed to evaluate performance while addressing gaps in knowledge related to: factors that successfully link the production of injury data with its use; and those factors that strengthen a system and support sustainability over time.

## Competing interests

The authors declare that they have no competing interests.

## Authors' contributions

AMA and TMD were members of the ACCISS evaluation team. AMA developed the research questions in collaboration with all co-authors and developed the manuscript. TMD substantially contributed to the development of the Figures. All authors contributed to the development of the manuscript. All authors read and approved the final manuscript.

## Pre-publication history

The pre-publication history for this paper can be accessed here:

http://www.biomedcentral.com/1471-2458/11/744/prepub
